# Novel Biomarkers, Including *tcdB* PCR Cycle Threshold, for Predicting Recurrent Clostridioides difficile Infection

**DOI:** 10.1128/iai.00092-23

**Published:** 2023-03-28

**Authors:** Gregory R. Madden, Isaura Rigo, Rachel Boone, Mayuresh M. Abhyankar, Mary K. Young, William Basener, William A. Petri

**Affiliations:** a Division of Infectious Diseases & International Health, Department of Medicine, University of Virginia School of Medicine, Charlottesville, Virginia, USA; b School of Data Science, University of Virginia School of Medicine, Charlottesville, Virginia, USA; c Department of Microbiology, Immunology, and Cancer Biology, University of Virginia, Charlottesville, Virginia, USA; University of California, Davis

**Keywords:** recurrent *Clostridioides difficile* infection, prediction model, risk model, outcome, medical outcomes

## Abstract

Traditional clinical models for predicting recurrent Clostridioides difficile infection do not perform well, likely owing to the complex host-pathogen interactions involved. Accurate risk stratification using novel biomarkers could help prevent recurrence by improving underutilization of effective therapies (i.e., fecal transplant, fidaxomicin, bezlotoxumab). We used a biorepository of 257 hospitalized patients with 24 features collected at diagnosis, including 17 plasma cytokines, total/neutralizing anti-toxin B IgG, stool toxins, and PCR cycle threshold (*C_T_*) (a proxy for stool organism burden). The best set of predictors for recurrent infection was selected by Bayesian model averaging for inclusion in a final Bayesian logistic regression model. We then used a large PCR-only data set to confirm the finding that PCR *C_T_* predicts recurrence-free survival using Cox proportional hazards regression. The top model-averaged features were (probabilities of >0.05, greatest to least): interleukin 6 (IL-6), PCR *C_T_*, endothelial growth factor, IL-8, eotaxin, IL-10, hepatocyte growth factor, and IL-4. The accuracy of the final model was 0.88. Among 1,660 cases with PCR-only data, cycle threshold was significantly associated with recurrence-free survival (hazard ratio, 0.95; *P < *0.005). Certain biomarkers associated with C. difficile infection severity were especially important for predicting recurrence; PCR *C_T_* and markers of type 2 immunity (endothelial growth factor [EGF], eotaxin) emerged as positive predictors of recurrence, while type 17 immune markers (IL-6, IL-8) were negative predictors. In addition to novel serum biomarkers (particularly, IL-6, EGF, and IL-8), the readily available PCR *C_T_* may be critical to augment underperforming clinical models for C. difficile recurrence.

## INTRODUCTION

A major challenge of Clostridioides difficile infection (CDI) is its tendency to reinfect despite antibiotic treatment. C. difficile colonizes the gut by producing spores that survive treatment and serve as a reservoir for recurrent growth of vegetative cells and reinfection, often related to microbiota disruption from anti-C. difficile or other antibiotics (1). Most recurrent CDI episodes are due to the previous strain (2–4) and typically occur within 2 to 8 weeks after the initial infection. Recurrence risk following an initial CDI episode is approximately 20% (5), a risk which increases upon subsequent infections (6), resulting in approximately 170,000 recurrent episodes of C. difficile infection annually in the United States (7).

Several new treatments are available that effectively prevent recurrent CDI, namely, fidaxomicin (8), bezlotoxumab (9), and fecal microbiota transplant (FMT) (10). Unfortunately, uptake of these interventions is poor (11) despite their adoption by consensus guidelines for CDI management (12), likely owing to their high costs (e.g., bezlotoxumab costs >$4,000 per vial [13] and the cost of fidaxomicin is 3× that of vancomycin [14]) and the lack of well-defined risk strata for recurrent C. difficile infection.

Accurately predicting which C. difficile infections will recur at the time of infection using conventional clinical factors has proven difficult. Existing clinical-only tools that attempt to stratify recurrence risk among CDI patients perform poorly on external validation, including at least 2 models that performed worse than chance (i.e., area under the receiver operating characteristic curve [AUROC], 0.42 to 0.43) (15).

Increasing evidence suggests that the host immune response is a critical factor that dictates future outcomes in CDI. For example, effective T-helper type 17 immune cells (defined by their interleukin 17 [IL-17] production) are required for the development of immunity against recurrent C. difficile infection (16). In addition, neutrophil-mediated inflammation, promoted by IL-8, is considered deleterious, while type 2 (or eosinophil-mediated) immunity is protective against severe C. difficile infection. Similarly, the C. difficile stool organism burden (which can be inversely approximated using the quantitative *tcdB* gene PCR cycle threshold [*C_T_*] [17]; i.e., low *C_T_* equals a high burden) has shown mixed associations with C. difficile severity (18); however, the utility of PCR *C_T_* for predicting recurrent C. difficile infection is unknown. In this study, we examined representative markers of C. difficile pathogenesis, including immune cytokines, anti-toxin antibodies, PCR *C_T_* (as a marker of stool organism burden), and stool toxins (A/B and binary toxin) (19) for predicting recurrent C. difficile infection using Bayesian machine learning.

## RESULTS

**FIG 1:**
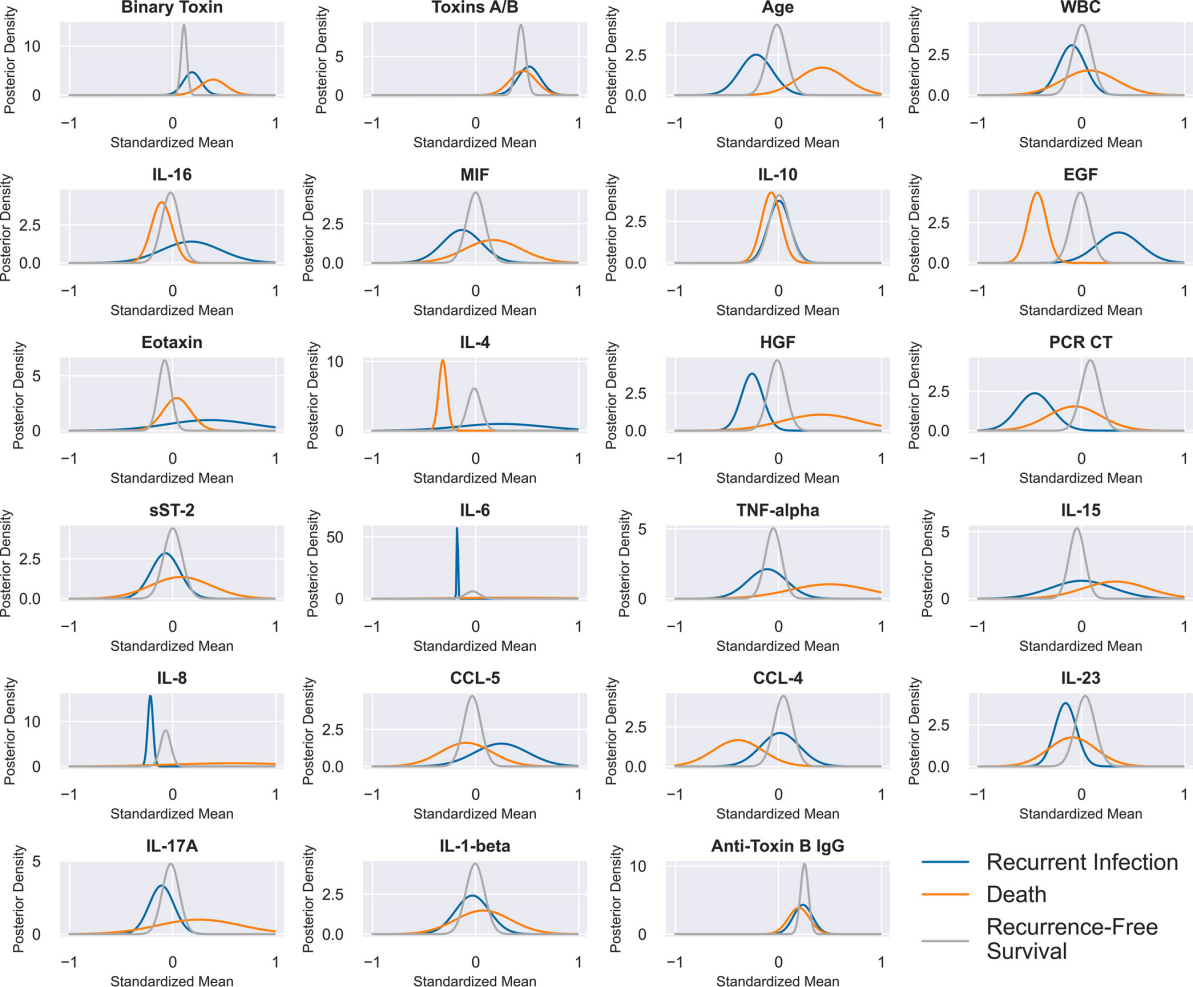
Marginal posterior probability distributions for the means of all variables (following standardization), given recurrence-free survival, death, or recurrent CDI within 90 days. We assume here that individual variables have Gaussian (normal) distributions with unknown means. Probabilities for the sample mean were then calculated over a range of possible values for each feature and class using Bayes’ theorem and plotted to represent a probability distribution, with its integral (or area under the curve) approximately equal to 1. The *y* axis can be interpreted as the relative likelihood that the mean of the sample was equal to the corresponding *x* value. For purposes of this analysis, 3 patients who both recurred and died within 90 days were factored as having died.

Biomarker data are summarized in Table 1, before preprocessing, with sample means separated based on patients who died, developed recurrent infection, or survived without recurrence. The posterior marginal probability density functions for the means of individual predictors, separated by 90-day outcome (recurrent CDI, CDI-associated mortality, recurrence-free survival) are shown in Fig. 1. For distributions that significantly overlapped (e.g., soluble ST-2 receptor [sST-2], IL-1b, anti-toxin B IgG), those variables by themselves would not useful for predicting that outcome. Lower age, PCR *C_T_*, IL-6, IL-8, and IL-17A and higher IL-16, EGF, and CCL-5 appeared to provide the best univariate class separation for recurrent infection (versus recurrence-free survival and death).

**TABLE 1:** Parameter means separated by C. difficile infection outcome^*a*^

Biomarker	Recurrence-free survival		Recurrent infection		Death	
	Mean	95% CI	Mean	95% CI	Mean	95% CI
Stool binary toxin	0.11	0.06–0.17	0.19	0.01–0.37	0.4	0.12–0.68
Stool toxins A/B	0.44	0.35–0.53	0.52	0.29–0.76	0.47	0.18–0.75
Age	60.64	57.78–63.5	57.38	51.98–62.78	67.93	59.55–76.32
WBC	13.07	11.61–14.52	12.28	10.06–14.49	13.66	8.88–18.44
IL-16	1,379.72	1,108.13–1,651.31	1,720.95	715.24–2,726.65	1,241.88	889.24–1,594.53
MIF	29,707.05	24,877.82–34,536.29	25,960.12	14,686.67–37,233.57	34,676.05	17,471.39–51,880.7
IL-10	8.32	3.21–13.42	8.26	2.25–14.26	6.27	0.77–11.76
EGF	270.55	221.91–319.19	374.72	251.17–498.27	157.44	102.98–211.9
Eotaxin	655.39	562.24–748.54	1,043.82	308.88–1,778.77	745.24	518.33–972.16
IL-4	94.75	68.3–121.21	159.0	0.00–355.59	32.63	14.94–50.32
HGF	638.91	502.18–775.64	440.51	258.38–622.64	1,037.39	313.9–1,760.89
PCR *C_T_*	26.65	25.75–27.54	23.85	22.01–25.69	25.83	22.82–28.84
sST-2	331,987.87	220,823.01–443,152.74	287,393.92	101,004.07–473,783.78	384,835.16	0.00–806,550.69
IL-6	46.17	11.41–80.94	8.61	5.47–11.75	182.93	0.00–530.5
TNF-α	9.51	8.12–10.9	8.95	5.32–12.59	15.12	6.87–23.37
IL-15	2.98	2.56–3.4	3.11	1.26–4.96	4.12	2.09–6.14
IL-8	162.87	111.46–214.27	85.73	57.79–113.66	657.21	0.00–1,468.79
CCL-5	50,205.12	43,689.06–56,721.19	61,880.04	39,509.3–84,250.79	47,687.87	25,303.24–70,072.51
CCL-4	2,151.66	2,022.28–2,281.04	2,129.27	1,831.44–2,427.09	1,814.72	1,416.71–2,212.73
IL-23	58.95	33.61–84.29	33.09	2.71–63.46	41.33	0.00–111.73
IL-17A	1.36	0.71–2.01	1.01	0.00–2.03	2.65	0.00–6.51
IL-1b	3.91	3.09–4.72	3.78	2.16–5.4	4.28	1.47–7.1
Anti-toxin B IgG	92.6	32.27–152.93	51.43	0.00–116.86	26.67	0.00–58.73
Toxin B neutralization	0.25	0.18–0.33	0.24	0.04–0.44	0.2	0.00–0.43

a Sample means are shown for raw (unscaled) parameter data in the subset of 163 observations with all available features, separated by patients who died (*n *= 15), developed recurrent infection (*n *= 21), or survived without recurrence (*n *= 127). Two-sided 95% confidence intervals (CIs) for the means were calculated using the Student’s *t*-distribution. For purposes of this analysis, 3 patients who both recurred and died within 90 days were factored as having died. Stool toxins and toxin B neutralization capacity were factored as 0 (negative) or 1 (positive), so their means represent the proportions of patients with positive results. WBC, white blood cell count; MIF, macrophage migration inhibitory factor; EGF, endothelial growth factor; HGF, hepatocyte growth factor; TNF-α, tumor necrosis factor-α.

Logistic regression with Bayesian model averaging was performed using all 24 features. Model averaging results are shown in Table 2. The most probable feature in the best-performing models for predicting recurrent CDI in a multivariable model was IL-6 (probability, 0.764), followed by PCR *C_T_* (0.711), EGF (0.192), IL-8 (0.111), eotaxin (0.066), IL-10 (0.058), hepatocyte growth factor (HGF) (0.056), and IL-4 (0.047).

**TABLE 2:** Bayesian model averaging results^*a*^

Biomarker	Probability	Avg coefficient
IL-6	0.7641	−10.4966
PCR *C_T_*^*b*^	0.7105	−0.4846
EGF	0.1922	0.0649
IL-8	0.1109	−0.1932
Eotaxin	0.0664	0.0130
IL-10	0.0582	0.0583
HGF	0.0560	−0.0224
IL-4	0.0465	0.0075
CCL-5	0.0438	0.0107
IL-23	0.0411	−0.0162
Stool toxins A/B	0.0350	−0.0108
Age	0.0339	−0.0090
IL-16	0.0314	0.0050
MIF	0.0211	−0.0017
Stool binary toxin	0.0185	0.0036
WBC	0.0173	0.0005
IL-1b	0.0173	−0.0035
sST-2	0.0142	0.0023
Anti-toxin B IgG	0.0113	−0.0026
IL-17A	0.0105	−0.0008
IL-15	0.0102	0.0003
TNF-α	0.0101	−0.0006
CCL-4	0.0101	−0.0003
Toxin neutralization	0.0101	0.0000

a The probability for each predictor variable represents the sum of the probabilities for all possible models containing that predictor variable. In other words, what is the chance that the “best” model (among possible biomarker combinations) will contain that biomarker? And if that biomarker is included, what is its regression coefficient (log odds), averaged across all models that contain it?

b PCR *C_T_*, C. difficile *tcdB* gene PCR cycle threshold.

**FIG 2:**
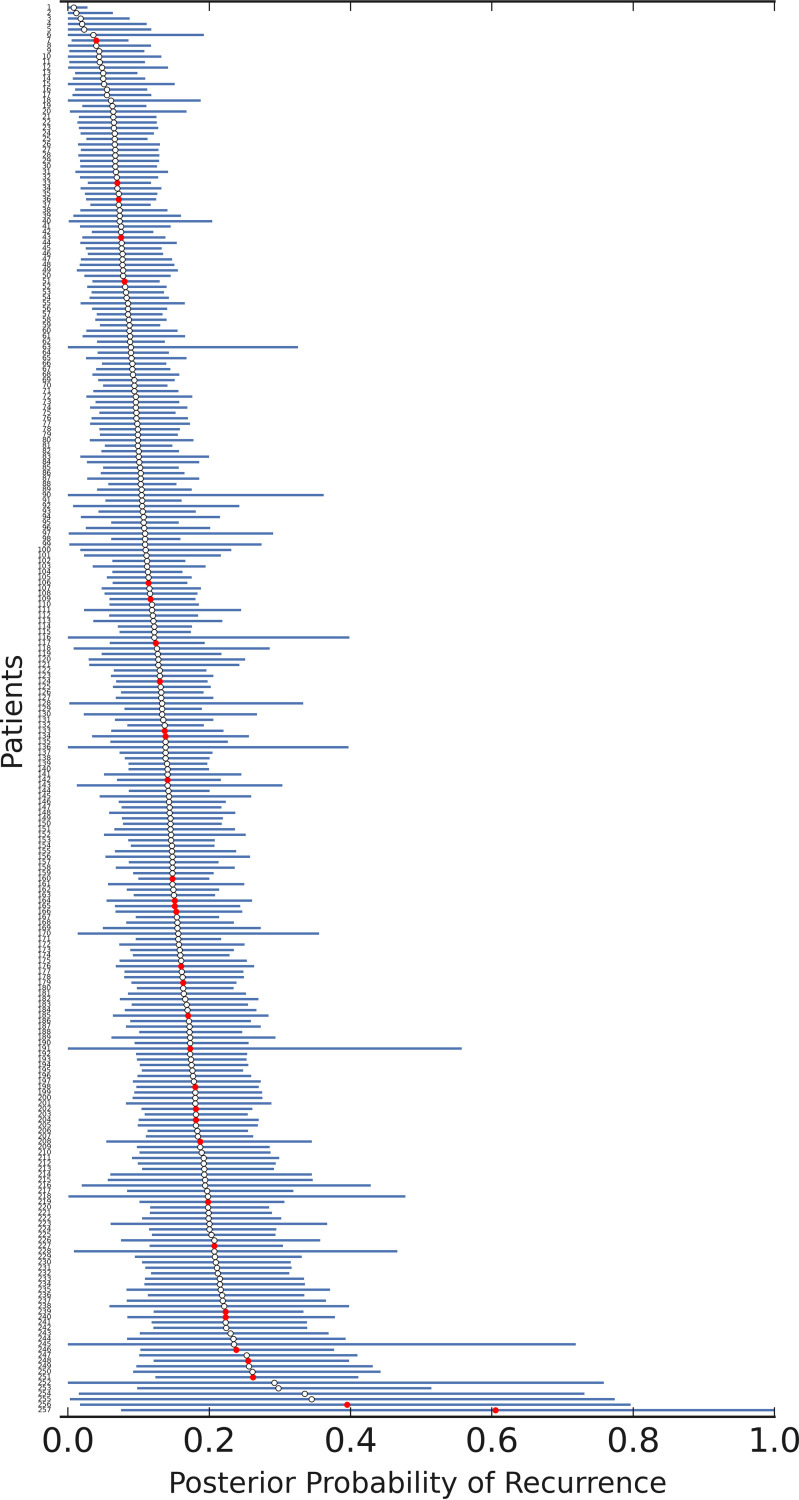
Mean posterior probabilities from combined traces (circles) for C. difficile recurrence risk for all 257 observations according to the Bayesian logistic regression model, with 95% credible intervals shown as horizontal lines. Actual observed 90-day recurrent CDI outcomes are represented by red circles.

**FIG 3:**
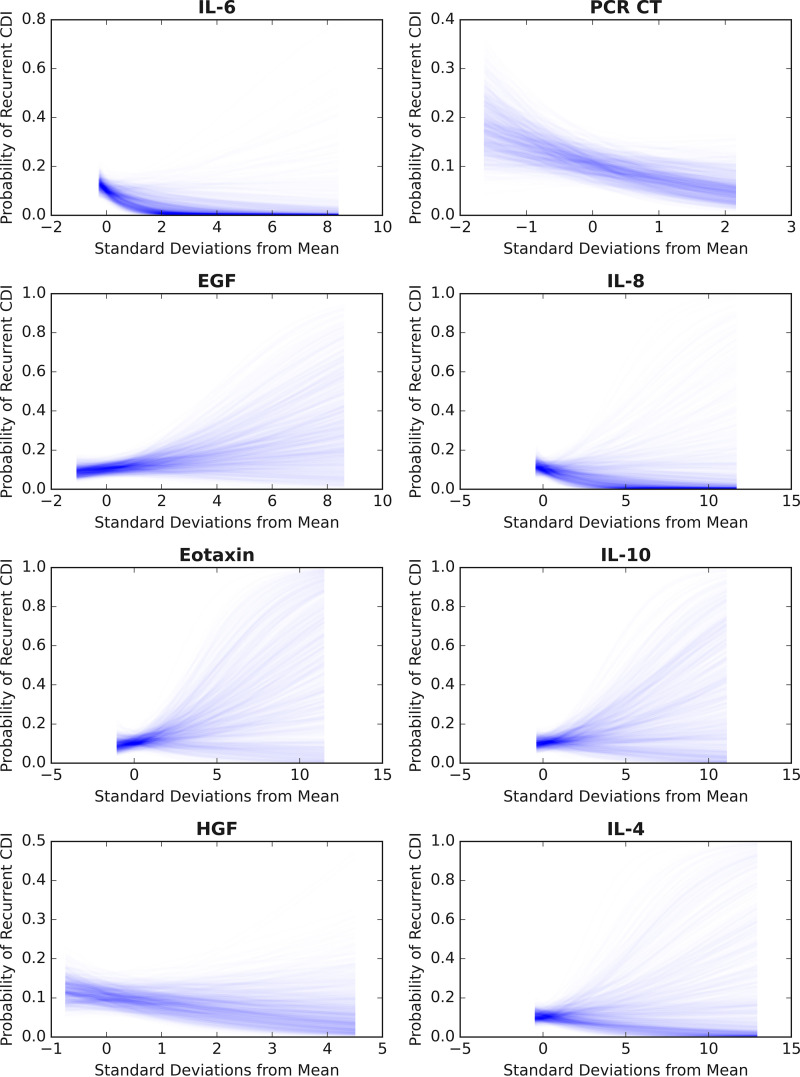
Individual biomarker effects on the posterior probability of recurrent C. difficile infection are shown by iteratively holding all other variables at their standardized means except for the query variable. To convey uncertainty around these estimates, 1,000 sample lines are plotted from the posterior predictive probabilities for each biomarker according to the fitted Bayesian logistic regression model.

The top 8 features, each with a probability of ≥0.05 based on model averaging, were used to train the final Bayesian logistic regression model using all 257 observations, including 32 recurrent CDI events (graphical model shown in Fig. S1 in the supplemental material). Markov chain Monte Carlo (MCMC) sampling (Metropolis-Hastings algorithm) was implemented to sample the posterior distributions of the coefficients using 30,000 samples, discarding the initial 15,000 samples as burn-in. Good convergence was seen with all coefficients (see trace plots in Fig. S2). The results of the final fitted Bayesian multivariable logistic regression model are shown in Table 3. Increased levels of IL-6, PCR *C_T_*, IL-8, HGF, and lower EGF, eotaxin, IL-10, and IL-4 were independently associated with lower adjusted odds of recurrent CDI. However, no singular feature was a statistically significant independent predictor in the presence of the other features. Sampling estimates of the posterior probabilities of individual patients are shown in Fig. 2. The AUROC of the final model was 0.66 (the ROC curve is shown in Fig. S3). To visualize the isolated effects of individual biomarker fluctuations on the risk of recurrence, we plotted posterior predictive regression lines for each biomarker while holding the other predictors constant at their standardized means (Fig. 3).

**TABLE 3:** Fitted Bayesian logistic regression model results

Biomarker	Mean adjusted odds ratio	95% credible interval
IL-6	0.43	0.14–1.15
PCR *C_T_*	0.69	0.46–1.03
EGF	1.15	0.86–1.58
IL-8	0.78	0.40–1.43
Eotaxin	1.18	0.79–1.76
IL-10	1.10	0.77–1.56
HGF	0.82	0.52–1.23
IL-4	0.99	0.65–1.49

**FIG 4:**
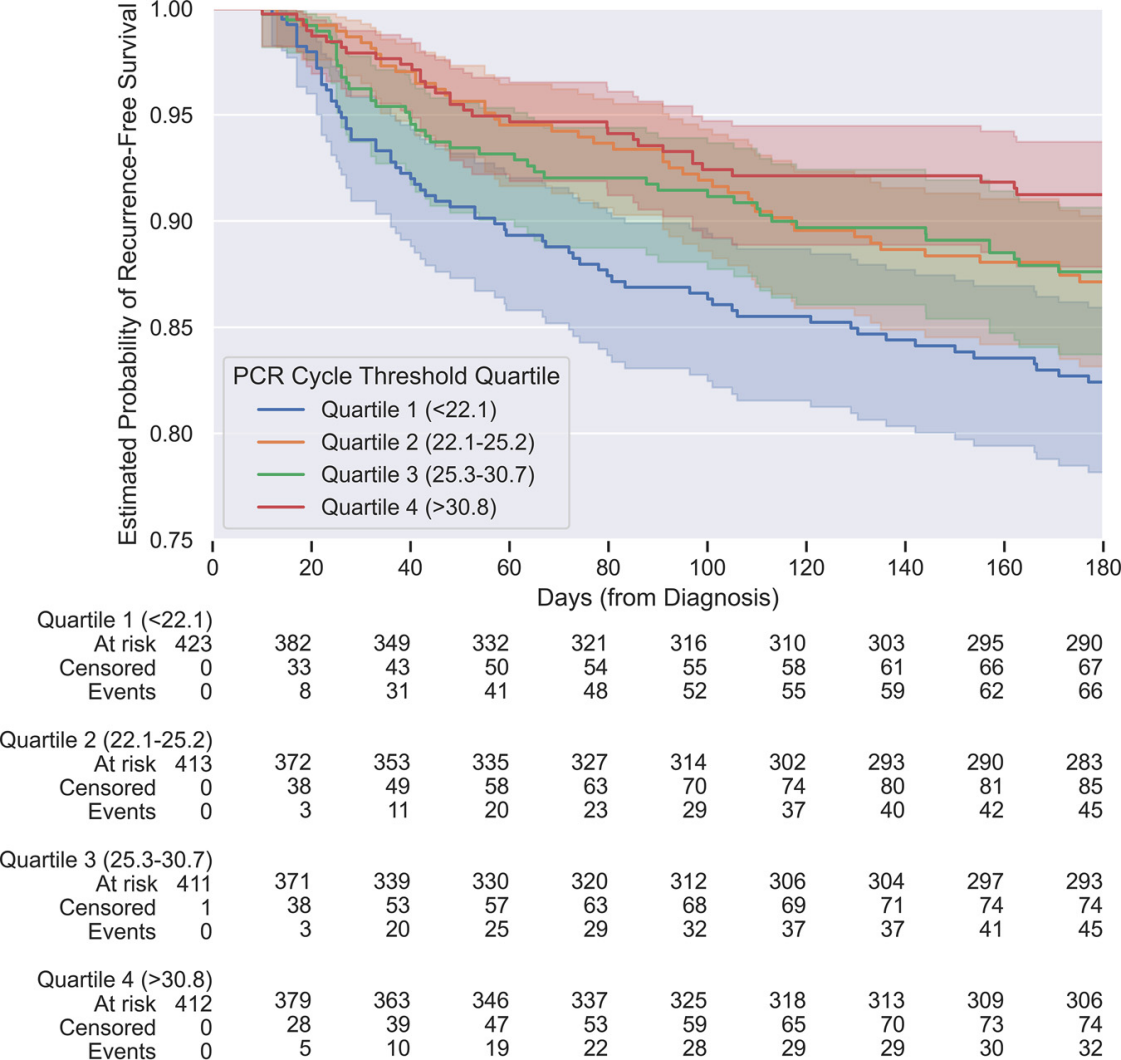
Kaplan-Meier curves for *tcdB* PCR cycle threshold quartiles and C. difficile recurrence-free survival. Shaded areas indicate 95% confidence intervals for each quartile.

For the PCR *C_T_* analysis, archived PCR data were available for 1,660 (of 2,126 total) hospitalized cases of C. difficile infection that occurred between November 2013 and April 2021 among 1,412 individual patients, of whom 250 (15.1%) suffered a recurrent episode of infection within 180 days. The PCR *C_T_* measurements ranged from 17.7 to 37.0 cycles. A Kaplan-Meier curve depicting recurrent C. difficile infections grouped by PCR *C_T_* quartiles is shown in Fig. 4. According to a univariate Cox regression, the hazard ratio for PCR *C_T_* was 0.95 (interpreted as a 5% relative lower risk of recurrent infection for each 1 standardized unit increase in PCR *C_T_*), which was statistically significant (95% confidence interval, 0.93 to 0.98; *P < *0.005). The AUROC for PCR *C_T_* alone for recurrent CDI within 90 days was 0.60 (Fig. S4). At the optimal Youden cutoff of ≤27.2 cycles, PCR *C_T_* achieved a sensitivity of 0.70, a negative predictive value of 0.89, a specificity of 0.43, and a positive predictive value of 0.18.

## DISCUSSION

The eight features identified as important by model averaging for predicting recurrent CDI can be classified into three groups: bacterial burden (PCR *C_T_*), type 17 immunity (IL-6, IL-8, IL-10), and type 2 immunity (EGF, eotaxin, HGF, IL-4). Considering the parameters with the largest effects on the posterior probability of recurrent infection (i.e., IL-6, PCR *C_T_*, EGF, IL-8), bacterial burden and type 2 immunity clearly emerged as positive predictors and type 17 immunity as a negative predictor of recurrent CDI. This is in contrast to our understanding that type 17 immunity is deleterious (20, 21) and type 2 immunity protective (22–24) against severe disease and C. difficile-associated mortality.

CDI recurrence is thought to represent a disruption in the process of inflammation, intestinal damage/repair, bacterial clearance, and the return to homeostasis. We hypothesize that type 17 effectors function as a double-edged sword, clearing C. difficile infection and reducing the risk of recurrence at the price of damaging intestinal tissue and contributing to disease severity (25). We also hypothesize that type 2 immunity, generally considered anti-inflammatory and a driver of epithelial healing (26, 27), may be reducing early gut damage but at the cost of not fully clearing the infection and increasing the opportunity for recurrence. Although we showed that early (within 48 h of diagnosis) type 2 immunity portends future recurrence, late type 2 activity is characteristic of a healed gut (27), with limited probability of recurrence (e.g., increased type 2 activity was shown 60 days post-successful FMT) (28).

Interestingly, the second highest probability factor for predicting recurrent CDI was not an immune biomarker, but the PCR *C_T_*. The negative coefficient for PCR *C_T_* suggests that a higher stool organism burden is associated with a higher risk of recurrence. Using a much larger, PCR-only data set, we found that *C_T_* performed remarkably well as a sole predictor of recurrent infection, possibly as a downstream indication of the balance between type 17 and type 2 immunity. PCR *C_T_* information is not widely accepted for use in the context of CDI management (12) but may be clinically underutilized. To date, low C. difficile PCR *C_T_* (reflecting high stool organism genomic equivalents) has been shown to predict toxin A/B enzyme immunoassay (EIA) (29) and cell cytotoxicity neutralization positivity (30), increased C. difficile-associated diarrhea/pain (31), longer duration of diarrhea (32), hypervirulent ribotype 027 (33), and disease severity/mortality (33, 34) (although other studies have failed to correlate *C_T_* with disease severity [35, 36]). Garvey et al. previously reported an association between low *C_T_* and treatment failure requiring a change of therapy or recurrent infection within 30 days (37), but to our knowledge, PCR *C_T_* has not been appreciated as an important predictor of recurrent infection. Quantitative PCR is used by >70% of U.S. hospitals (38), and because *C_T_* values are calculated for all clinical PCR results at CDI diagnosis, *C_T_* could theoretically be employed relatively easily by itself or alongside other markers to better define risk. Other traditional markers for severe CDI (age, white blood cell count [WBC], stool toxin A/B positivity [39]) were found to be unimportant for recurrence alongside the other biomarkers. We also showed that the presence of stool binary toxin (which is produced by 027 and other hypervirulent strains) was not associated with recurrent infection; this observation runs counter to one theory that increasing rates of recurrence are related to the emergence of hypervirulent strains (6).

C. difficile toxin B alone has emerged as the main virulence factor in CDI. Previous studies have demonstrated that patients who lack a robust anti-toxin B IgG response following infection are more likely to develop recurrent disease (40–42); however, these findings have not always been consistent, likely due to the heterogeneity of methods and early versus late timing when measuring humoral immunity (43, 44). We showed that the early (within 48 h) anti-toxin B IgG response (perhaps reflecting prior colonization or more advanced infection) was nonpredictive for recurrence, but it may be a strictly late marker of protection. Similarly, bezlotoxumab (a monoclonal antibody against toxin B) did not significantly affect early outcomes (symptom duration, severity, mortality) in a randomized placebo-controlled trial (mean time to antibody, 3 days from start of treatment) (45), and subsequent trials failed to demonstrate consistent benefits of anti-toxin antibodies in treating acute infection (46).

Unlike predicting CDI severity, where a multitude of clinical severity scoring systems exist with reasonable performance, such as ATLAS (47, 48), there are currently no well-established tools to predict the recurrence of C. difficile infection. We used a naive Bayesian approach to evaluate a rich set of novel predictors shown to be important in CDI pathogenesis (19, 20). The performance of our biomarker-based model for C. difficile recurrence was comparable to the best-performing clinical-based model described by D’Agostino et al. (AUROC, 0.64) (49); however, there is clearly room for further improvement. Bayesian model averaging allowed us to objectively quantify the probabilities of individual predictors to include in our final model. One issue with traditional (frequentist) methods of prediction is that individual point estimates of risk can be unknowingly overconfident. We used Bayesian inference because it allows researchers and clinicians to better account for uncertainty when forecasting C. difficile recurrence. Credible intervals of our posterior estimates varied considerably from patient to patient, highlighting the importance of a probabilistic approach to understanding patient outcomes.

This study has limitations. Our list of measured serum cytokines was chosen based on our knowledge of C. difficile immunopathogenesis but is not exhaustive. In the Bayesian logistic regression model, no single predictor was statistically significant in the presence of the other predictors, suggesting that all the biomarkers must be present or that there was an insufficient sample size. The rates of recurrent C. difficile infection in both cohorts (32/257 [12.8%], biorepository; 250/1,660 [15.1%], PCR only) were relatively low compared with some reports in the literature, which could be explained by incomplete capture of recurrent cases (which required patients to be diagnosed within the same health system laboratory). It is also possible that some recurrent CDI episodes detected by PCR were in fact reflective of asymptomatic C. difficile colonization (which ranges from 3% to 21% in the general population [50]). While our institution has a history of robust diagnostic stewardship and clinical decision support to reduce inappropriate C. difficile testing that could help to minimize this issue (51, 52), it remains an important caveat. Missing data were primarily due to insufficient discarded stool or sera (or *C_T_* data that failed to archive, in the case of the PCR analysis), which was presumably introduced randomly; however, missing data bias could not be excluded. Finally, all performance measures were within-sample and do not guarantee generalizability; further studies are needed to validate PCR *C_T_* alone and alongside other novel biomarkers before they can be adopted for routine clinical use.

## MATERIALS AND METHODS

### Stool and cytokine measurements.

Details of the UVA CDI biorepository and serum cytokine and stool toxin assays have been previously published (19, 20). At the University of Virginia Health (645-bed, tertiary-care academic hospital) between 2013 and 2016, discarded stool and sera were collected from hospitalized adult patients within 48 h of a diagnosis of CDI, excluding those diagnosed with CDI in the preceding 90 days. Serum cytokine measurements were obtained using Luminex assays (R&D Systems, Minneapolis, MN), with the exceptions of sST-2, IL-23, and IL-17A, which were measured using an enzyme-linked immunosorbent assay (ELISA). Stool toxin A/B and binary toxin were detected using qualitative ELISA (TechLab, Blacksburg, VA). Basic clinical, PCR, and outcome data (i.e., age, white blood cell count, mortality, recurrence) were collected retrospectively from the electronic medical record.

### Anti-toxin B humoral immunity.

Anti-toxin B antibodies were quantified using ELISA, whereby 96-well plates were coated overnight with 1 μg/mL of purified toxin B (TechLab). The plates were incubated and washed, and goat anti-human IgG and Fcγ fragment specific antibody (Jackson Laboratory; 109-035-098) were added, followed by TMB substrate (Thermo Scientific; 34028). Optical densities were read at 450 nm, and endpoint titers were interpolated using a cutoff optical density of 0.1. The antitoxin neutralization capacity was determined using a cell cytotoxicity neutralization assay. Serially diluted sera (1/40 to 1/2,560) were incubated with purified toxin B (TechLab; diluted to 1 ng/mL) for 1 h at 37°C; the mixtures were then incubated with CHO-K1 cells (ATCC CCL-61) for 24 h and inspected by light microscopy to determine cytopathic effects. Neutralization capacity was determined if any serum dilution protected 100% of CHO-K1 cells.

### Bayesian logistic regression.

Quantitative measurements (age, white blood cell count [WBC; max available within 48 h], PCR *C_T_*, IL-4, IL-6, IL-8, IL-10, IL-15, IL-16, IL-23, IL-1β, C-C chemokine ligand-4 [CCL-4], CCL-5, hepatocyte growth factor [HGF], soluble ST-2 receptor [sST-2], tumor necrosis factor-α [TNF-α], macrophage inhibitory factor [MIF], endothelial growth factor [EGF], eotaxin, anti-toxin B immunoglobulin G [IgG] titers) were standard scaled (mean, 0; standard deviation, 1) using all available data. A feature selection step was performed using a subset of 163 patients with all 24 features available. Ordinary logistic regression was performed with Bayesian model averaging using the Occam’s window method (model growing where models with a probability of >1/20th of the top model are chosen) and constant priors. After selecting the highest probability features from model averaging (using the Bayesian information criterion), we implemented a Markov chain Monte Carlo (MCMC) sampling estimate for the posterior distributions of the coefficients of a final Bayesian logistic regression model using Gaussian priors.

### PCR *C_T_* and recurrence-free survival analysis.

For the PCR-only analysis, all inpatient qualitative PCR results coupled with available quantitative archived cycle threshold values were collected from the clinical microbiology laboratory GeneXpert PCR machine (Cepheid, Sunnyvale, CA) between November 2013 and April 2021 (in addition to follow-up positive recurrent CDI results within at least 180 days). Hospitalized index CDI episodes were defined as the first available positive PCR result accompanied by anti-C. difficile treatment (with oral vancomycin, metronidazole, or fidaxomicin) within the ensuing 10 days, and recurrent infection was defined as a repeat positive PCR result occurring >10 days after the index infection. Hospital medication administration and mortality data were collected from the electronic medical record. A Cox proportional hazards regression model was developed to measure the effect of PCR *C_T_* on days to recurrent CDI, whereby death was treated as a censoring event. Analyses were performed using Python version 3.9.13 and the following packages: Statsmodels (Bayesian model averaging) (53), GraphViz (graphical model) (54), pymc3 (MCMC sampling) (55), Scikit-learn (performance metrics, ROC analysis) (56), and lifelines (Cox regression, Kaplan Meier analysis) (57).

### Ethics statement.

Collection of patient samples (biorepository cohort) and analysis of clinical data (including from the *C_T_* data cohort) were approved by the University of Virginia Institutional Review Board (IRB-HSR 16926 and 20082, respectively), with waivers of informed consent.
