# Supplementary material for: Novel Biomarkers, Including tcdB PCR Cycle Threshold, for Predicting Recurrent Clostridioides difficile Infection
Source: Infect Immun. 2023 Mar 28;91(4):e00092-23. doi: 10.1128/iai.00092-23 (PMC10112139; doi:10.1128/iai.00092-23)
Supplement: Supplemental file 1 — Fig. S1 to S4. Download iai.00092-23-s0001.pdf, PDF file, 1.1 MB [file iai.00092-23-s0001.pdf]

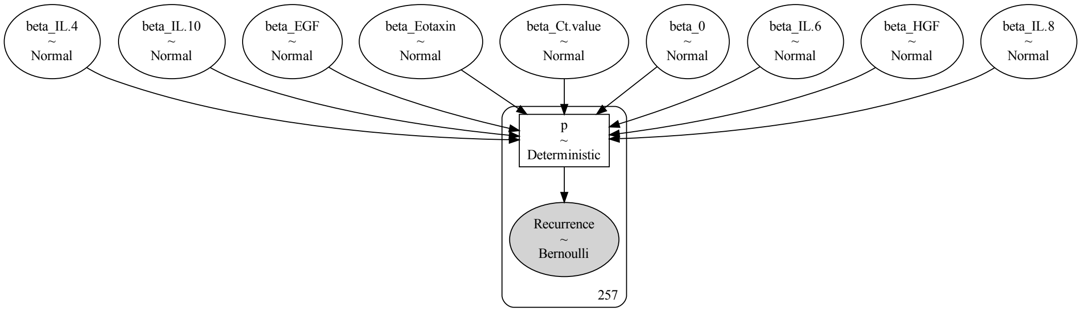

Supplementary FIG 1. Graphical Model of the Bayesian Logistic Regression Model.

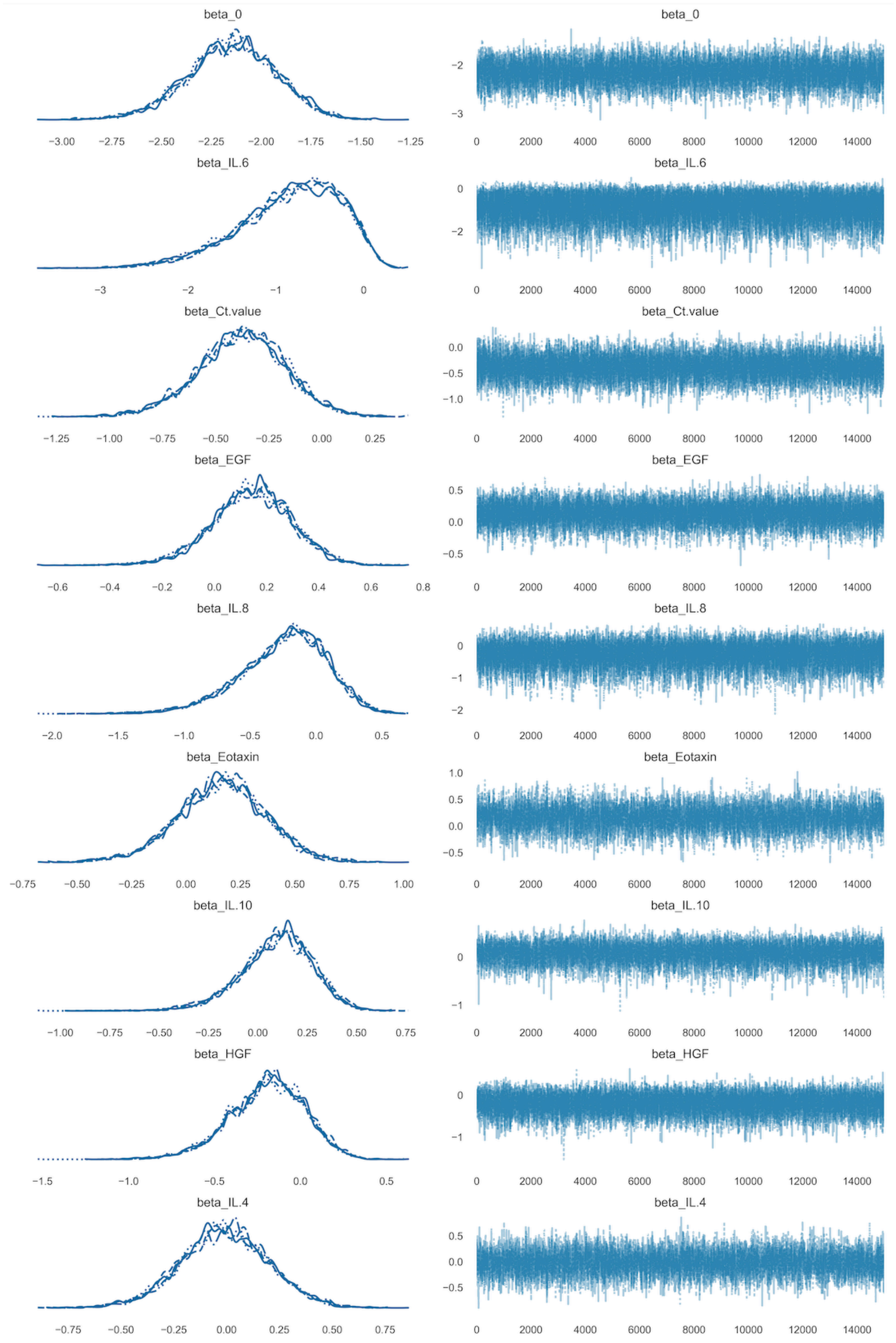

Supplementary FIG 2. Markov chain Monte Carlo (MCMC) Sampling Traceplots (4 chains). MCMC sampling produces very close fits to the posterior distributions but is computationally expensive and may not be appropriate for very large datasets (Raftery et al. 1997. J Am Stat Assoc 92:179–191). The smaller size of our dataset was not prohibitive in this regard.

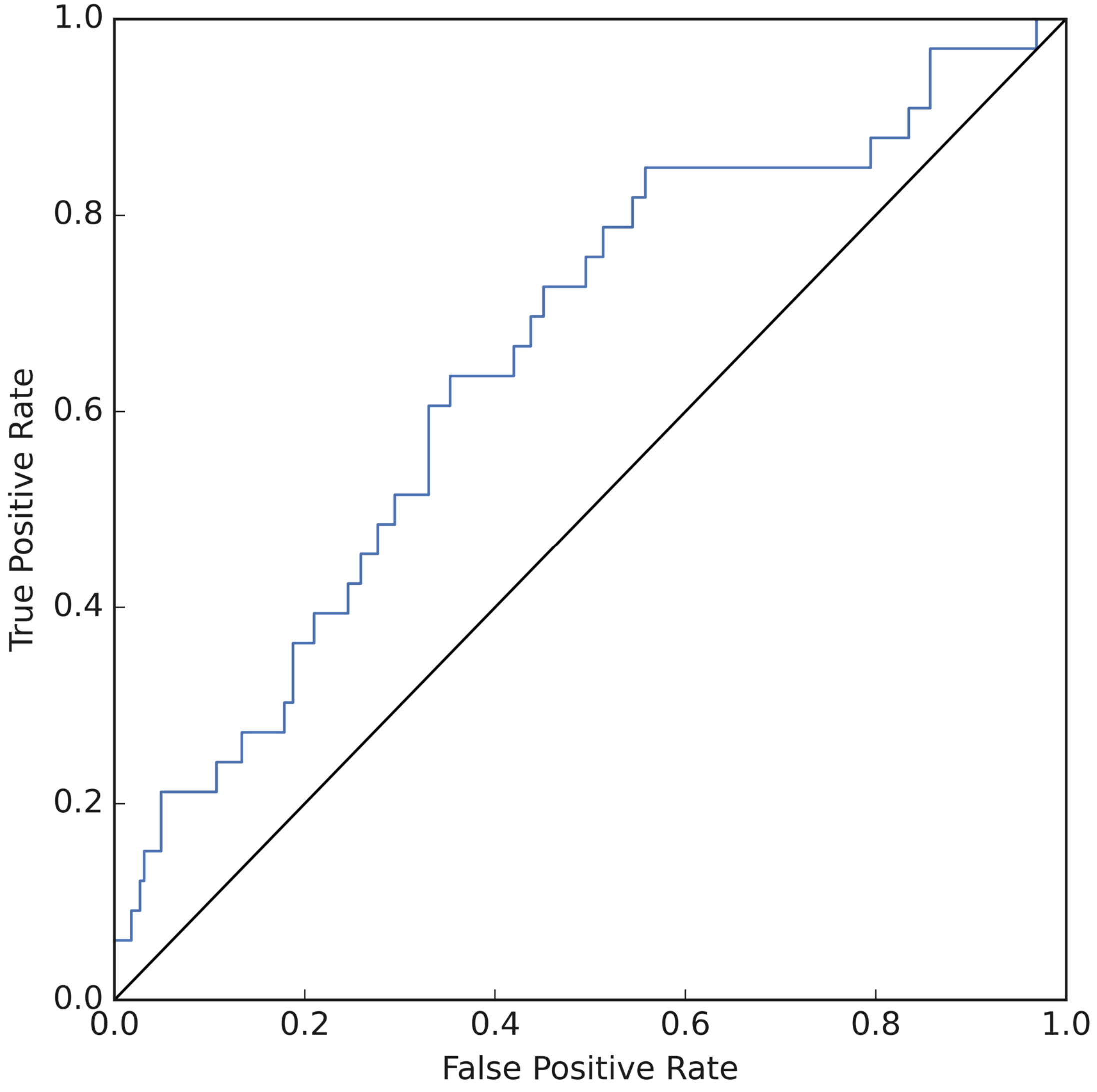

Supplementary FIG 3. Receiver operating characteristic curve for the *C. difficile* Recurrence Bayes Logistic Regression Model**.** Area under the curve is 0.66.

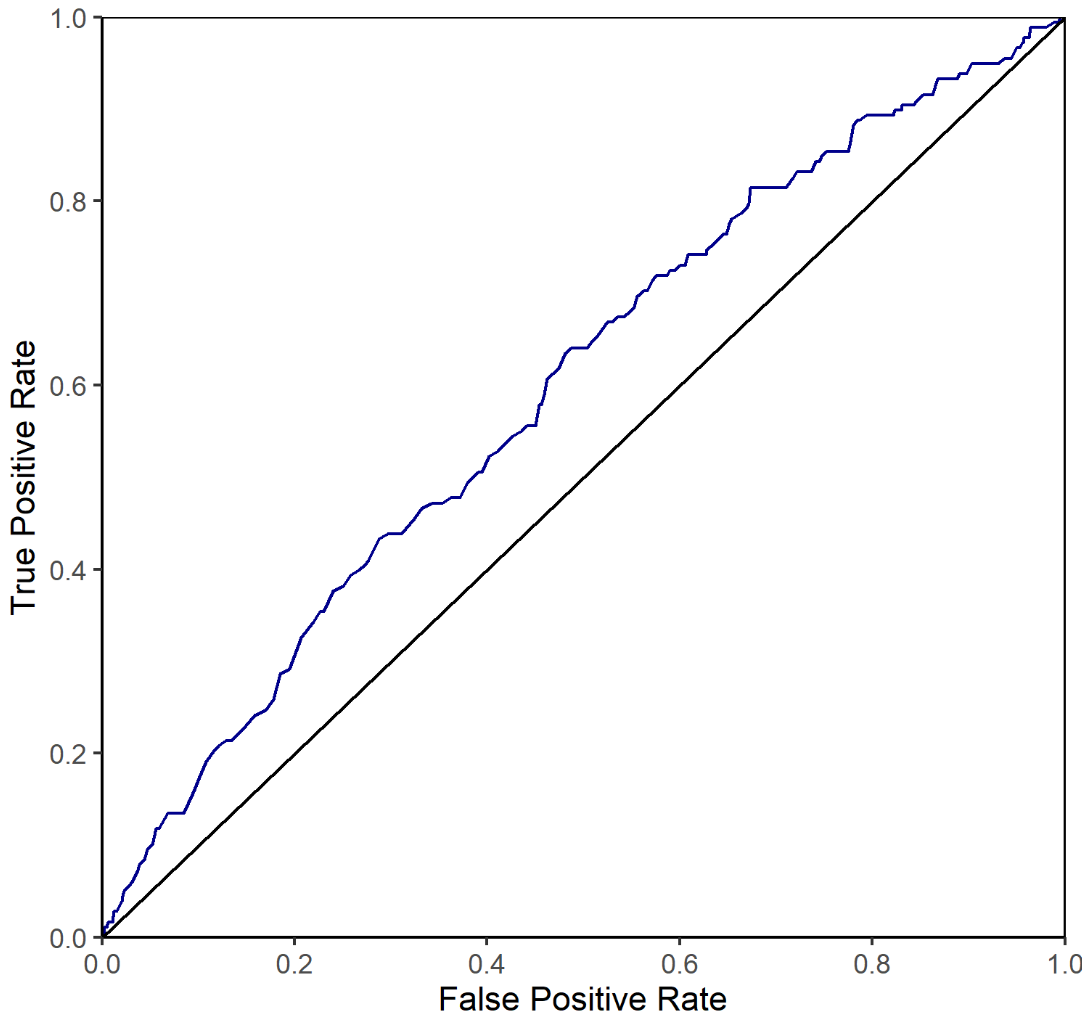

Supplementary FIG 4. Receiver operating characteristic curve for PCR CT alone for recurrent *C. difficile* infection within 90 days. Area under the curve is 0.60.
